# The Relationship Between Knowledge and Practice of Postreproductive Women Toward Prevention and Screening of Breast and Cervical Cancer in Saudi Arabia

**DOI:** 10.7759/cureus.44858

**Published:** 2023-09-07

**Authors:** Fatmah Alsharif, Fathia Ibrahim, Munirah Alotaibi, Maha Khalaf, Hajer Algaidi, Ohood Felemban, Mai Yaseen

**Affiliations:** 1 Medical and Surgical Nursing, King Abdulaziz University, Jeddah, SAU; 2 Public Health Nursing, King Abdulaziz University, Jeddah, SAU; 3 Nursing, King Abdulaziz University, Jeddah, SAU; 4 Public Health, King Abdulaziz University, Jeddah, SAU

**Keywords:** cervical cancer, saudi women, knowledge, prevention, breast cancer

## Abstract

Introduction

Cervical cancer and breast cancer are the major causes of mortality among women worldwide, and the burden of cancer incidence is increasing exponentially. The aim of this study was to assess the relationship between knowledge and practice of postreproductive women toward prevention and screening of breast and cervical cancer in Saudi Arabia.

Methods

A quantitative, descriptive, cross-sectional study was conducted using a convenience sampling method. One hundred and twenty-eight participants completed the online survey. The questionnaire consists of four main sections: sociodemographic data, obstetrical history, knowledge, and practice of breast and cervical cancer. The correlation coefficient and chi-square test were used to analyze the data.

Results

Nearly 40% of the participants had good knowledge of the risk factors of breast cancer; 80% had good knowledge of early warnings of breast cancer; 66% had fair knowledge of prevention measures of breast cancer; and 68% had good knowledge of prevention measures of breast cancer. Only 23% of participants had poor knowledge of risk factors of cervical cancer, whereas 62% had fair knowledge of early signs and early screening methods of cervical cancer. The majority of the respondents (85%) had good knowledge of prevention measures for cervical cancer; however, less than one-third of the participants (31%) and 39% had poor or fair practice regarding screening and prevention of breast and cervical cancer, respectively. A significant relationship between practice and knowledge was found as well as a significant relationship between practice and educational level as the p-value was less than 0.05.

Conclusion

Despite having a comprehensive understanding of avoidable malignancies and screening methods, postreproductive women’s utilization of breast and cervical cancer examinations was inadequate. Hence, continuous awareness programs are needed to help women modify their habits and early detections.

## Introduction

Cervical cancer and breast cancer are the most frequent cancers among women worldwide [1]. 2.3 million new instances of breast cancer were diagnosed worldwide in 2020, and 685, 000 women died as a result of it. More than 7.8 million women had been diagnosed with breast cancer in the previous five years, making it the most common cancer type worldwide. Disability-adjusted life years (DALYs) from breast cancer were likewise higher than those from any other type of cancer in women, as it was the leading cause of these DALYs. The incidence of breast cancer is higher in later life. However, it can strike women of any age after puberty anywhere in the world [2]. Breast cancer is the most common malignancy in Saudi women, accounting for 21.8 percent of all cases in the country. As the population ages and grows, breast cancer mortality among Saudi women is likely to climb in the next decades according to the most recent study of cancer-related mortality among Saudi women. Saudi Arabia's King Faisal Specialist Hospital and Research Center's Saudi Cancer Registry estimates that 930 new instances of breast cancer are diagnosed per year. When it comes to cancer diagnoses among women in Saudi Arabia, breast cancer is the most common, accounting for 27.4 percent of the 5,378 cases in 2010 [3]. As cervical cancer is considered the fourth most frequent cancer in women globally, it is expected to see 604, 000 new cases and 342,000 deaths in 2020 [1]. In Saudi Arabia, according to a recent statistic, 358 new cervical cancer cases and 179 deaths occur annually which makes cervical cancer the eighth most common cancer among women between the ages of 15 and 44 [4]. Breast and cervical cancer development is influenced by a wide range of risk factors; becoming older is the most significant risk factor. As a result, postreproductive women are twice as likely to be diagnosed with cancer due to hormonal factors [5].

Literature review 

A literature review was conducted to assess previous literature on women's knowledge and attitude toward breast and cervical cancer worldwide. In India, a cross-sectional survey of 200 female healthcare workers revealed that 26.5 percent and 7 percent of respondents conducted clinical breast examinations and mammograms respectively. Over 90% of those surveyed were aware of risk factors, symptoms, and screening instruments available for cervical and breast cancer. A full understanding of the preventable malignancies and screening procedures, however, was not enough to provide sufficient screening of breast and cervical cancers [6]. Another study in India included five rural villages in Tamil Nadu's Vellore district which enrolled women aged 25-60. Findings revealed that 43.8% and 57.9% were aware of the availability of cervical and breast cancer screening, respectively. Sufficient knowledge of breast cancer (score of 50% or higher) was only 5.9%, and cervical cancer was 27.2% [7]. 

In Zimbabwe, one of the several studies that included 409 persons revealed that most of those polled (almost 85 percent) have heard of cancer, 34.2 percent were uninformed of any risk factors for cervical cancer, and 51 percent were unaware of the signs and symptoms associated with cervical cancer. The vast majority of those polled (96.2%) had never undergone screening for cervical cancer [8].

In Egypt, Beni-Suef University also conducted a study with the participation of 500 students. There is a shortage of information about breast and cervical cancers; students notably poorly understood both types of cancer screening [9]. Another study was conducted in Turkey on 668 women, and all of the women had an insufficient frequency of screening tests [10].

Additional research in Nigeria and Egypt examining 1006 women revealed that "lack of awareness of where to get screened" and "lack of symptoms" were the main explanations for low screening uptake [11]. Another study that enrolled 799 women ages 18 and over revealed that women poorly understood breast and cervical cancer risk factors [12].

In Ethiopia, a cross-sectional investigation of 770 participants revealed that cervical cancer was mentioned by more than half of the participants (65.1%). The majority of the studied sample (more than 80 percent) is not aware that HPV is the primary cause of cervical cancer. Only 107 (21.4 percent) of those who had heard of the Papanicolaou stain test were aware of it. Less than half of the studied sample (43.9 percent) believes that women who appear to be in good health should be tested at least three times throughout their lifetime. In all, 153 people (19.87 percent) had enough knowledge about cervical cancer and prevention. Cervical cancer awareness among women was low [13].

In Iraq, a cross-sectional research study conducted in Erbil city on 416 women aged between 20 and 60 revealed that 25.5 percent of females had performed breast self-examination and 15.9 percent had undergone mammography. Additionally, one-third of the studied sample stated that they were unaware of how to perform breast self-examination. Cervical cancer awareness was poor (23.3%), as were attitudes toward it (56%) and the use of the Papanicolaou smear as a screening tool (10.1%). The likelihood of a Pap smear examination by a healthcare worker was higher (16.5 percent vs. 9.2 percent). Respondents said that 69 percent of them had not been advised to obtain a Pap test by their doctor. Only 7.5% of women were aware of the HPV vaccine [14].

The literature reviewed in this study concluded that women aged 20 to 60 either have poor knowledge regarding breast and cervical cancer and few percentages are aware of and take the screening tests. However, there has been little research on postreproductive-age women's understanding and practice of breast and cervical cancer screening and prevention to this point. The purpose of this study is to determine the knowledge and practice of postreproductive age women regarding the prevention and screening of breast and cervical cancer in Saudi Arabia.

## Materials and methods

Research design and setting 

A quantitative, descriptive, cross-sectional study design was carried out in Saudi Arabia. The research was conducted among women above 45 years old from March 2020 to April 2020. To make it easier, data were collected via an online self-reported questionnaire using a Google Drive website. The link was distributed to potential participants via social media, including Twitter and WhatsApp groups.

Sampling and sample size 

Because of the descriptive nature of the study, the convenience sampling technique was used to collect the necessary data. The questionnaire targeted all women who live in Saudi Arabia. The inclusion criteria were women who were above 45 years old and Saudi; the exclusion criteria were women who were below 45 years old or non-Saudi. Based on the G*power program, with an alpha of 0.05 and a power of 0.95, the total sample size of 128 had adequate statistical power. Hence, a minimum sample size of 128 was required for data collection. 

Instrumentation

Participants were given a detailed questionnaire after they accepted participation. The questionnaire was composed of four main sections. In the first section, we obtained sociodemographic data of participants, such as religion, marital status, living place, educational level, socioeconomic status, and employment. In the second section, we collected obstetrical history, for example, regulation of the menstrual period, number of children, pregnancy and delivery, age of marriage, and first pregnancy. In the third and fourth sections, we collected questions about knowledge and practice of cervical and breast cancer, including 46 items regarding risk factors, early warning signs, early screening, and prevention measures. A questionnaire was developed by the researchers and revised by five experts in the field of nursing from the faculty staff to test face and content validity. The reliability of the questionnaire was tested on several levels. Pilot testing of the questionnaire was performed where 19 participants were recruited to investigate the ability of the respondents to complete the questionnaire and assess the clarity of the questionnaire and the need to add or delete certain items. To test reliability, we conducted internal consistency via a Cronbach’s alpha test of the questionnaire, which was well acceptable because Cronbach’s alpha of the total scale was 0.766. In addition, the responses to these questions included yes-or-no, multiple-choice, and open-ended questions. The questions were prepared in the English language and translated into Arabic by two experts for better understanding by women.

Data collection and procedures 

The data collection was investigated by distributing a questionnaire. Questionnaires were selected for this study because they are a reliable and fast way to collect data from a large number of people within a short period. The questionnaire included sociodemographic data, obstetrical history, knowledge, and practice of breast and cervical cancers. The participants were approached online using social media, including WhatsApp, Snapchat, and Twitter. The questionnaire was distributed using an online Google Forms tool. The target population was informed about the purpose and scope of the research. Additionally, ethical considerations were also taken to ensure the confidentiality and privacy of the collected data.

Data analysis 

To achieve the aims of the study and analyze the collected data, many appropriate statistical methods were carried out using IBM SPSS Statistics for Windows, Version 28 (Released 2021; IBM Corp., Armonk, New York, United States). Then, the following statistical measures were calculated: frequencies and percentages, to understand the characteristics of the participants and determine their responses to the main axis items that were included in the study tool, and weighted mean, to know the average responses of the participants on each of the axis items, which is useful for arranging the items according to the highest weighted average. A weighted mean from 0 to less than 0.33 indicates a poor level of knowledge, from 0.33 to less than 0.66 indicates a fair level of knowledge, and from 0.66 to 1 indicates a good level of knowledge. Arithmetic mean and standard deviation helped us to understand the extent of the high or low responses of the participants and the extent of the deviation of the responses of the participants for each of the items of the study variables, respectively. The correlation coefficient and chi-square test were used to measure the degree of correlation between two variables. The value of this parameter expresses the strength of the relationship between the two variables, and some approximate indicators can be used to judge the degree of this relationship. If the value of the correlation coefficient lies between 0 and 0.5, this indicates the weakness of the relationship, whereas if its value lies between 0.5 and 1, this indicates the strength of this relationship. The relationship between the two variables is absent if the correlation coefficient is zero, whereas the value of one for the correlation coefficient indicates a complete relationship between the two variables. The sign of the correlation coefficient usually indicates the direction of the relationship between the two variables.

Ethical considerations 

The study was approved by the ethical and research committee of the Faculty of Nursing Jeddah at King Abdulaziz University in Saudi Arabia (NREC Serial No.: Ref. No. 2B. 39). The study was wholly voluntary, and the data collection was completely anonymous so that the participants’ identities would not be exposed. There was comprehensive information about the study on the first page of the online survey instrument. As a result, participants had the opportunity to read the material offered before beginning the survey and decide whether or not to participate. Thus, the completion and submission of the survey imply the participants’ informed permission.

## Results

The questionnaire was distributed among women living in Saudi Arabia. A total of 128 responses were collected and used in the analysis. The findings in Table 1 show that all of the studied samples were Muslim and 75% of them were married. This table also shows that the majority of women live in Jeddah (79.7%) and more than half of the total (55.5%) have a university education degree. Regarding their economic status, the majority (87.5%) have enough salary.

**Table 1:** Distribution of the studied samples according to sociodemographic characteristics

Sociodemographic characteristics		Number (n=128)	%
religion	Muslim	128	100.0%
Marital status	Married	96	75.0%
	Single	11	8.6%
	Widow	7	5.5%
	Divorced	14	10.9%
Living place	Jeddah	102	79.7%
	Riyadh	6	4.7%
	Taif	1	0.8%
	Yanbu	1	0.8%
	Mecca	7	5.5%
	Medina	3	2.3%
	Eastern Province	2	1.6%
	Abha	1	0.8%
	Jazan	1	0.8%
	Dammam	2	1.6%
	Qassim	1	0.8%
	Khobar	1	0.8%
Educational level	Illiterate	2	1.6%
	Primary	2	1.6%
	Intermediate	9	7.0%
	Secondary	35	27.3%
	University	71	55.5%
	Higher education	9	7.0%
Economic status	Not-enough	16	12.5%
	Enough	112	87.5%
Employment	Non employed	73	57.0%
	Employed	55	43.0%

The results from Table 2 present the knowledge of the studied sample regarding risk factors of breast cancer. It is clear from the results that the studied samples have a good knowledge of smoking as a risk factor for breast cancer, followed by age > 45 years (74% and 73%, respectively). However, the majority of the studied samples (93%) did not know that hormone replacement therapy (HRT) is a risk factor for breast cancer.

**Table 2:** Distribution of the studied samples according to their knowledge of risk factors regarding breast cancer

Risk factors of breast cancer	No		Yes		Mean	Standard deviation	Level of knowledge
	No	%	No	%			
Smoking	33	25.8%	95	74.2%	0.74	0.44	Good
Age > 45 years	35	27.3%	93	72.7%	0.73	0.45	Good
Stress	49	38.3%	79	61.7%	0.62	0.49	Fair
Oral hormonal contraceptive use	71	55.5%	57	44.5%	0.45	0.50	Fair
Obesity	75	58.6%	53	41.4%	0.41	0.49	Fair
Exposure to radiation therapy	80	62.5%	48	37.5%	0.38	0.49	Fair
Less physical activity	92	71.9%	36	28.1%	0.28	0.45	Poor
Family history of breast cancer	99	77.3%	29	22.7%	0.23	0.42	Poor
Previous history of breast cancer	115	89.8%	13	10.2%	0.10	0.30	Poor
Hormone replacement therapy (HRT)	119	93.0%	9	7.0%	0.07	0.26	Poor
Total					0.40	0.15	

Table 3 indicates the distribution of the studied samples according to their knowledge of early signs of breast cancer. It is clear from the results that the studied samples have good knowledge concerning the presence of breast lumps, change in skin color, change in the shape of the breast, and abnormal discharge from the nipple as early signs of breast cancer (85.2%, 82.0%, 81.3%, 78%, and 72%, respectively).

**Table 3:** Distribution of the studied samples according to their knowledge about early warning signs of breast cancer

Early signs of breast cancer	No		Yes		Mean	Standard Deviation	Level of knowledge	order
	No	%	No	%				
Breast lump	19	14.8%	109	85.2%	0.85	0.36	Good	1
Change in skin color and shape of breast	23	18.0%	105	82.0%	0.82	0.39	Good	2
Abnormal discharge from nipple	24	18.8%	104	81.3%	0.81	0.39	Good	3
Change in the color or shape of a woman's nipple	28	21.9%	100	78.1%	0.78	0.42	Good	4
Pain in breast	36	28.1%	92	71.9%	0.72	0.45	Good	5
Total					0.80	0.31	Good	

Table 4 shows that the studied samples have good knowledge of preventive measures for breast cancer, where the studied samples have good knowledge of evidenced exposure to radiation preventing breast cancer and abstaining from smoking prevents breast cancer (77.3% and 71.9%, respectively), whereas more than half of the studied samples have fair knowledge that adequate sleep prevents breast cancer, with a mean of 0.54. 

**Table 4:** Distribution of the studied sample according to their knowledge about preventive measures of breast cancer

Preventive measures of breast cancer	No		Yes		Mean	Standard Deviation	status	order
	No	%	No	%				
Does avoiding exposure to radiation prevent breast cancer?	29	22.7%	99	77.3%	0.77	0.42	Good	1
Does abstaining from smoking prevent breast cancer?	36	28.1%	92	71.9%	0.72	0.45	Good	2
Periodic examination	41	32.0%	87	68.0%	0.68	0.47	Good	3
Does breast feeding prevent breast cancer?	43	33.6%	85	66.4%	0.66	0.47	Good	4
Does physical activity prevent breast cancer?	48	37.5%	80	62.5%	0.63	0.49	Fair	5
Does healthy diet prevent breast cancer?	51	39.8%	77	60.2%	0.60	0.49	Fair	6
Does enough sleep prevent breast cancer?	59	46.1%	69	53.9%	0.54	0.50	Fair	7
Total					0.66	0.30	Good	

Table 5 shows that the studied samples have good knowledge of early screening methods for breast cancer. The majority of the studied samples (82.8%) have good knowledge regarding breast self-examination as a screening measure, where they have fair knowledge (58.6%) about screening methods using mammograms, with a mean of 0.59.

**Table 5:** Distribution of the studied samples according to their knowledge of early screening of breast cancer

Early screening of breast Cancer	No		Yes		Mean	Standard Deviation	Level of knowledge
	No	%	No	%			
Is there a need to do a breast self-examination?	22	17.2%	106	82.8%	0.83	0.38	Good
Have you heard of mammogram?	48	37.5%	80	62.5%	0.63	0.49	Fair
Is there a need to do mammogram screening?	53	41.4%	75	58.6%	0.59	0.49	Fair
Total					0.68	0.32	Good

Table 6 illustrates that the studied samples have poor practice related to screening for breast cancer. More than half (52%) of the studied samples mentioned they had never done a BSE before. The table also shows that about one-third of the studied samples had undergone mammograms before.

**Table 6:** Distribution of the studied samples according to their practice regarding breast cancer screening

Practice regarding breast cancer screening	Not done		Done	
	No	%	No	%
Have you done a breast self-examination before?	67	52.3%	61	47.7%
Have you ever been screened undergone mammogram before?	88	68.8%	40	31.3%
Have you performed breast self-examination regularly?	108	84.4%	20	15.6%

Table 7 illustrates the knowledge of the studied samples concerning the risk factors of cervical cancer. The results show that the studied samples have poor knowledge regarding the family history of cancer, a history of STIs, and infection with HPV (30%, 5%, and 5%, respectively). However, up to half of the studied samples (52%) had a fair knowledge of having weak immunity being a risk factor.

**Table 7:** Distribution of the studied samples according to their knowledge of risk factors regarding cervical cancer

Risk factors of cervical cancer	No		Yes		Mean	Standard Deviation	Level of knowledge
	No	%	No	%			
Having a weakened immunity (such as the diagnosis of chronic diseases, use of cortisone	61	47.7%	67	52.3%	0.52	0.50	Fair
Family history of cancer	89	69.5%	39	30.5%	0.30	0.46	Poor
Having history of STI	122	95.3%	6	4.7%	0.05	0.21	Poor
Infection with human papilloma virus	122	95.3%	6	4.7%	0.05	0.21	Poor
Total					0.23	0.22	Poor

Table 8 illustrates the studied sample’s knowledge about early signs of cervical cancer, where 70% of the studied samples have good knowledge that abnormal vaginal discharge is a sign of cervical cancer and 64% have fair knowledge of abnormal vaginal bleeding and pain during sex as a sign of cervical cancer.

**Table 8:** Distribution of the studied samples according to their knowledge of early signs of cervical cancer

Early signs of cervical cancer.	No		Yes		Mean	Standard Deviation	Level of knowledge
	No	%	No	%			
Abnormal vaginal discharge	38	29.7%	90	70.3%	0.70	0.46	Good
Abnormal vaginal bleeding	46	35.9%	82	64.1%	0.64	0.48	Fair
Pain during sex	46	35.9%	82	64.1%	0.64	0.48	Fair
Total					0.62	0.42	Fair

Table 9 represents the knowledge of the studied samples regarding early screening measures of cervical cancer. Slightly less than three quarters (74%) of the studied samples have good knowledge that a Pap smear is considered as an early screening measure for cervical cancer. In addition, more than half (63%) have fair knowledge about the pelvic sonar as a screening measure. 

**Table 9:** Distribution of the studied samples according to their knowledge of early screening measures of cervical cancer

Early screening measures of cervical cancer	No		Yes		Mean	Standard Deviation	Level of knowledge
	No	%	No	%			
Pap smear	33	25.8%	95	74.2%	0.74	0.44	Good
Is Pap smear done only after the age of 45 ?	34	26.6%	94	73.4%	0.73	0.44	Good
Pelvic sonar	47	36.7%	81	63.3%	0.63	0.48	Fair
Do you know what are the methods for early detection of cervical cancer?	80	62.5%	48	37.5%	0.38	0.49	Fair
Total					0.62	0.31	Fair

Table 10 demonstrates the practice regarding screening and prevention of cervical cancer. More than half of the studied samples (57.0%) have never undergone a Pap smear. Also, only 1.6% of the studied sample had the HPV vaccination. However, 71.1% of the studied sample reported they checked with a doctor if they felt any infections in the reproductive system or a change in secretion.

**Table 10:** Distribution of the studied samples according to their practice regarding screening and prevention of cervical cancer

Practice regarding screening and prevention of cervical cancer	Not Done		Done	
	No	%	No	%
Do you check with a doctor if you feel any infections in the reproductive system or a change in secretions?	37	28.9%	91	71.1%
Have you ever been screened (pap smear) before?	73	57.0%	55	43.0%
Have you ever had the HPV vaccination?	126	98.4%	2	1.6%

Table 11 indicates the distribution of the studied samples according to their knowledge about preventive measures of cervical cancer. It is clear from the table that the studied samples have good knowledge about Pap smears and periodic examination (85.9% and 83.6%, respectively).

**Table 11:** Distribution of the studied samples according to their knowledge about prevention measure of cervical cancer

Prevention Measure of cervical Cancer	No		Yes		Mean	Standard Deviation	Level of knowledge
	No	%	No	%			
Pap smear	18	14.1%	110	85.9%	0.86	0.35	Good
Periodic examination	21	16.4%	107	83.6%	0.84	0.37	Good
Total					0.85	0.33	Good

Table 12 shows that there is a significant relation between practice and knowledge (p-value <.05).

**Table 12:** Correlations between total knowledge and practice Statistically significant at p < 0.05

	Practice		
	Pearson Correlation	Sig. (two-tailed)	N
Knowledge	.351^**^	0.000	128

Table 13 shows that there is no significant relation between knowledge and the sociodemographic characteristics.

**Table 13:** Relation between total knowledge and sociodemographic characteristics The p-value of the chi-square test greater than 0.05.

		know_cat							
		Poor		Fair		Good		chi-square	sig.
		Count	%	Count	%	Count	%		
Marital status	Married	5	3.9%	50	39.1%	41	32.0%	3.784	0.706
	Single	1	0.8%	8	6.3%	2	1.6%		
	Widow	0	0.0%	4	3.1%	3	2.3%		
	Divorced	0	0.0%	8	6.3%	6	4.7%		
Living place	Jeddah	3	2.3%	58	45.3%	41	32.0%	24.556	0.219
	Riyadh	1	0.8%	2	1.6%	3	2.3%		
	Taif	0	0.0%	0	0.0%	1	0.8%		
	Yanbu	0	0.0%	0	0.0%	1	0.8%		
	Mecca	1	0.8%	5	3.9%	1	0.8%		
	Medina	0	0.0%	2	1.6%	1	0.8%		
	Eastern Province	1	0.8%	0	0.0%	1	0.8%		
	Abha	0	0.0%	1	0.8%	0	0.0%		
	jazan	0	0.0%	1	0.8%	0	0.0%		
	Dammam	0	0.0%	0	0.0%	2	1.6%		
	Qassim	0	0.0%	1	0.8%	0	0.0%		
	Khobar	0	0.0%	0	0.0%	0	0.0%		
Educational level	Illiterate	1	0.8%	1	0.8%	0	0.0%	14.110	0.168
	Primary	0	0.0%	2	1.6%	0	0.0%		
	Intermediate	1	0.8%	6	4.7%	2	1.6%		
	Secondary	1	0.8%	18	14.1%	16	12.5%		
	University	3	2.3%	38	29.7%	30	23.4%		
	Higher education	0	0.0%	5	3.9%	4	3.1%		
Socioeconomic status	Not-Enough	1	0.8%	10	7.8%	5	3.9%	0.695	0.706
	Enough	5	3.9%	60	46.9%	47	36.7%		
Employment	Non employed	3	2.3%	41	32.0%	29	22.7%	0.223	0.895
	Employed	3	2.3%	29	22.7%	23	18.0%		

Table 14 shows no significant relation between practice and sociodemographic characteristics, where the p-value of the chi-square test was greater than 0.05. However, a significant relationship between practice and educational level was found, where the p-value of the chi-square test was less than 0.05. 

**Table 14:** Relation between practice and sociodemographic characteristics * Statistically significant at p < 0.05

		practice_cat				chi-square	sig
		Non done		Done			
		Count	%	Count	%		
Marital status	Married	68	40.0%	55	32.4%	1.890	0.596
	Single	13	7.6%	5	2.9%		
	Widow	5	2.9%	4	2.4%		
	Divorced	11	6.5%	9	5.3%		
Living place	Jeddah	79	46.5%	59	34.7%	8.987	0.623
	Riyadh	4	2.4%	4	2.4%		
	Taif	0	0.0%	1	0.6%		
	Yanbu	0	0.0%	1	0.6%		
	Mecca	6	3.5%	2	1.2%		
	Medina	2	1.2%	1	0.6%		
	Eastern Province	1	0.6%	1	0.6%		
	Abha	1	0.6%	1	0.6%		
	jazan	1	0.6%	0	0.0%		
	Dammam	2	1.2%	0	0.0%		
	Qassim	0	0.0%	1	0.6%		
	Khobar	0	0.0%	1	0.6%		
Educational level	Illiterate	4	2.4%	1	0.6%	11.959	.035^*^
	Primary	2	1.2%	2	1.2%		
	Intermediate	7	4.1%	7	4.1%		
	Secondary	39	22.9%	13	7.6%		
	University	38	22.4%	43	25.3%		
	Higher education	7	4.1%	7	4.1%		
Socioeconomic status	Not-Enough	18	10.6%	10	5.9%	0.715	0.398
	Enough	79	46.5%	63	37.1%		
Employment	Non employed	55	32.4%	40	23.5%	0.061	0.804
	Employed	42	24.7%	33	19.4%		

## Discussion

To the best of our knowledge, this study is one of the fewer studies that assess the knowledge and practice of postreproductive age women toward prevention and screening of breast and cervical cancer in Saudi Arabia.

In this study, we found that nearly 40% of the studied samples have a fair knowledge of risk factors of breast cancer. They had the least knowledge of the risk factor and it was HRT with a ratio of 7%. However, it is clear from the results that the studied samples have a good knowledge that smoking has a risk factor of 74.2%. Inconsistent with the literature, a study done by Saha and colleagues among rural women in Vellore, Tamil Nadu revealed that they have (12%) knowledge about risk factors of breast cancer [7]. However, it is known that rural women have always less knowledge and healthcare accessibility compared with urban women. Another finding in this study is that participants have knowledge about breast cancer symptoms revealing that a lump in the breast (85.2%) and a change in skin color and breast shape (82%) are warning signs of breast cancer. Our finding is in contrast with the rural Zimbabwe study when Over 18% of respondents did not know of any breast cancer symptoms [8]. Low knowledge could be due to low coverage of cancer awareness initiatives in African countries. This calls for action to improve the knowledge of breast cancer. Also, in this study, we found that 68% of the studied samples have a good knowledge of early screening for breast cancer. It is clear from the results that they have a good knowledge of breast self-examination. As for the mammogram, it was the lowest. Consistent with the literature, a study of 1006 women in Egypt and Nigeria showed good knowledge of early screening (breast self-examination and mammogram) with a proportion of 68.59% [11].

This study reveals that less than half of the studied samples have poor knowledge regarding the risk factors of cervical cancer (23%). However, despite their poor knowledge, they had fair knowledge about having weak immunity considering a risk factor for cervical cancer with an average of 52.3%. It was also clear from the results that they have less knowledge of Infection with human papillomavirus. This finding is consistent with a study in Northwest Ethiopia and their study showed that around 40% had poor knowledge of the risk factor of cervical cancer [13]. Nearly 62 % of the studied samples had fair knowledge of early warning signs of cervical cancer. The results indicated that abnormal vaginal discharge is the most familiar to them. In contrast, the result is higher than that in a study conducted in Northwest Ethiopia, which shows that only 19.96% of the studied sample were aware of the symptoms of cervical cancer [13]. This difference is probably due to higher education levels of participants and better cervical cancer awareness programs in the latter countries. Another finding is that about 71.1% of the participants claimed that they check with a doctor if they feel any infection in the reproductive system or change in secretion. Only 2 (1.6%) respondents have ever been vaccinated with the HPV vaccine. Consistent with the literature, poor vaccine uptake was also found among 251 Indian women where only 19 (7.6 %) received the HPV vaccines. The reason cited in that study was that the high cost of the HPV vaccines has been a major factor in not receiving the vaccines [15].

Overall, this study revealed that there is a significant relationship between knowledge and practice among postreproductive women, and when assessing knowledge and practice with demographic variables, the educational level of participants was found to be a significant practice variable. However, the current study shows that women generally have good knowledge about breast and cervical cancer. Although they are knowledgeable, they do not perform BSE or follow-up by regular mammograms with 84% and 68.8, respectively. Almost half of the studied samples (57%) has never undergone a Pap smear test. In this respect, our results are similar to the study conducted in Turkey which found that 52.4% of women between the age of 45-54 do not implement BSE [10]. Furthermore, low practice of the Pap smear test is also reported in this study (83.8%). This result may be due to people feeling that when they do not have symptoms, they do not need to make any medical checkup. Women in western Turkey showed that BSE and Pap testing decreased significantly with increased age [10]. Increasing knowledge of the importance of breast cancer screening annually after the age of 45 is considered a priority. Another study was conducted among women in Ethiopia, where less than 5% of women had ever undergone a Pap smear examination for cervical cancer screening [12]. 

Limitations

Our survey’s main limitations are the use of Internet-based questionnaires and the sample size. We had to use Internet-based questionnaires to determine the results due to the COVID-19 pandemic. Furthermore, participants may be lacking in technical knowledge. As a result, this limited our investigation to persons who had access to the Internet. This could have influenced the respondent’s choice of the Internet as their primary source of information. As a result, the findings cannot be applied to the entire Saudi Arabian population. Also, one of the biggest challenges we faced was the short time frame, which was a challenge for us. The absence of preceding research and participant misunderstandings of several questions further limited the study.

Strengths

This study has been extended to all regions of the Kingdom of Saudi Arabia. Of the studies published recently, 24% were recently published (2020, 2021). This included women from different backgrounds in rural and urban areas.

Recommendations

Development of community awareness programs to improve postreproductive women’s knowledge and practice regarding screening and prevention of breast and cervical cancers.

## Conclusions

Breast and cervical cancers are curable if detected early, which leads to a greater rate of survival. Cancer screening services help promote early detection and improve prognosis. This study showed good knowledge about breast cancer and cervical cancer in postreproductive women. However, this knowledge did not correlate with the use of screening services because the majority of the women surveyed had never been screened or vaccinated. The implementation of BSE and Pap smears was also poor. This requires further efforts to promote cancer screening and HPV vaccination. The media and the Internet are the primary sources of information for most women regarding the importance of screening and should be used more effectively. Leaflets provide information on and a clear description of the testing process and should be considered a priority. Further research should be directed at postreproductive women due to their vulnerability.
